# Prognostic Nutritional Index as a Novel Biomarker for Predicting Prognosis in Sepsis‐Associated Encephalopathy: A Multicenter Retrospective Cohort Study

**DOI:** 10.1155/emmi/4486190

**Published:** 2026-01-07

**Authors:** Lina Zhao, Chao Qi, Qinghe Yan, Yuehao Shen, Dongxue Huang, Haiying Liu, Xuguang Li, Yun Li, Keliang Xie

**Affiliations:** ^1^ Department of Critical Care Medicine, Tianjin Medical University General Hospital, Tianjin, 300052, China, tjmugh.com.cn; ^2^ Department of Anesthesiology, Nankai University Affiliated Beichen Hospital, Tianjin, 300400, China; ^3^ Department of Anesthesiology, The Second Hospital of Tianjin Medical University, Tianjin, 300211, China, tijmu.edu.cn; ^4^ Department of Anesthesiology, Tianjin Institute of Anesthesiology, Tianjin Medical University General Hospital, Tianjin, 300052, China, tjmugh.com.cn

**Keywords:** 28-day mortality, Glasgow Coma Scale, Prognostic Nutritional Index, sepsis-associated encephalopathy

## Abstract

**Background:**

Sepsis‐associated encephalopathy (SAE) has a high mortality rate with limited prognostic biomarkers. We investigated the relationship between the Prognostic Nutritional Index (PNI) and SAE outcomes.

**Methods:**

This multicenter cohort study (2008–2019) enrolled 3202 SAE patients. The primary outcome was 28‐day all‐cause mortality. Multivariable‐adjusted analyses (logistic regression, propensity score matching, and inverse probability weighting) assessed PNI’s prognostic value, supplemented by generalized additive models (GAMs), Kaplan–Meier, and ROC analyses. External validation was performed.

**Results:**

PNI independently predicted 28‐day mortality (adjusted OR: 0.85; 95% CI: 0.77–0.93). The GAM identified PNI = 34 as the optimal prognostic threshold. Patients with PNI < 34 had higher 28‐day mortality than those with PNI ≥ 34 in both original and validation cohorts (*p* < 0.001). ROC analysis demonstrated strong discrimination in the original cohort (AUC = 0.879; sensitivity = 0.878; specificity = 0.880) and the validation cohort (AUC = 0.724). Higher PNI correlated with better neurological function (Glasgow Coma Scale, *p* < 0.001).

**Conclusions:**

This multicenter study establishes the PNI as an independent predictor of 28‐day mortality in patients with SAE. We identified that SAE patients with PNI < 34 exhibited significantly higher 28‐day mortality rates and worse neurological function.

## 1. Introduction

Sepsis‐associated encephalopathy (SAE) is clinically defined as diffuse cerebral dysfunction secondary to systemic inflammatory responses in sepsis patients, in the absence of direct central nervous system (CNS) infection [1]. Previous studies have shown that the incidence of SAE is as high as 50%–70% [2, 3]. Critically, progression to SAE elevates sepsis mortality rates to approximately 50%, a marked increase compared to septic patients without encephalopathy [2, 3]. Currently, there is still a lack of effective indicators or biomarkers to evaluate the prognosis of patients with SAE. Therefore, it is particularly important to explore the early evaluation of the prognosis of SAE patients and to provide effective interventions to reduce the mortality rate of SAE patients.

The Prognostic Nutritional Index (PNI), calculated as serum albumin (g/dL) × 10 + total lymphocyte count (× 10^9^/L) × 5, integrates two pivotal pathophysiological pathways in SAE: Hypoalbuminemia reflects persistent systemic inflammation and blood–brain barrier disruption, while lymphopenia indicates immunosuppression and impaired microbial clearance [4–6]. Serum albumin, an endogenous neuroprotective agent, binds endotoxins and free radicals that contribute to neuroinflammation, and lower serum albumin levels were independently associated with increased 90‐day mortality in SAE patients [7]. In patients with delirium, higher albumin levels were associated with shorter hospital stays [8]. Concurrently, lymphocyte depletion correlates with secondary infections and failure to resolve systemic inflammation, both known drivers of SAE progression [9, 10].

This dual biomarker synergy explains that PNIs may have superior prognostic performance over isolated measures. We hypothesize that the PNI demonstrates superior prognostic accuracy compared to the conventional Sequential Organ Failure Assessment (SOFA) or Simplified Acute Physiology Score II (SAPS II) score in predicting the prognosis of SAE patients. In this multicenter study, we evaluate the prognostic value of the PNI for 28‐day mortality in patients with SAE, and the independent validation cohorts validate the results of the exploration.

## 2. Materials and Methods

### 2.1. Study Settings

This research leveraged the open‐source medical information from the Medical Information Mart for Intensive Care IV (MIMIC‐IV v2.0) database and the multicenter database electronic Intensive Care Unit Collaborative Research Database (eICU‐CRD v2.0). MIMIC‐IV is a comprehensive repository that encompasses patient data from Beth Israel Deaconess Medical Center, spanning the years 2008–2019 [11]. The eICU‐CRD aggregates ICU admission data from 208 U.S. hospitals during 2014–2015, representing a large‐scale multicenter cohort of critically ill patients. The study protocol was reviewed and approved by the institutional review board (IR No. 33690380), with all researchers completing mandatory human subject protection training through the collaborative institutional training initiative program. The above databases have been approved by the Massachusetts Institute of Technology Review Committee. The raw data were extracted by employing Structured Query Language (SQL) with Navicat and further processed using the R software.

### 2.2. Patients

All enrolled patients met the Sepsis‐3 diagnostic criteria [12]. SAE was defined by Glasgow Coma Scale (GCS) < 15 or delirium in the presence of sepsis [13–16]. For patients undergoing sedation or surgery, GCS scores before sedation or surgery were extracted. We excluded patients with the following conditions: (1) primary neurological conditions that could independently impair consciousness (traumatic brain injury, acute ischemic/hemorrhagic stroke, active epilepsy, or intracranial infection); (2) preexisting severe organ dysfunction (Child–Pugh Class C liver cirrhosis or end‐stage renal disease requiring dialysis); (3) acute life‐threatening comorbidities (postcardiac arrest status); (4) substance abuse disorders (chronic alcoholism or illicit drug use documented in medical records); (5) severe metabolic derangements (hyponatremia < 120 mmol/L, persistent hyperglycemia > 180 mg/dL, or hypoglycemia < 54 mg/dL despite correction); (6) insufficient observation time (ICU death/discharge within 24 h of admission); (7) incomplete neurological assessments (missing GCS documentation); (8) unavailable nutritional–inflammatory data (missing albumin or lymphocyte counts for PNI calculation); and (9) age < 18 years.

### 2.3. Data Collection

We collected comprehensive clinical data, including the following: (1) demographic characteristics (age and sex); (2) 28‐day mortality outcomes; (3) comorbidities classified using the International Classification of Diseases, Ninth Revision (ICD‐9) criteria; (4) mean vital sign measurements prior to SAE diagnosis; (5) mean laboratory measurements prior to SAE diagnosis and first available laboratory results following ICU admission; and (6) infection sites and causative microorganisms. Disease severity was assessed using standardized scoring systems recorded within 24 h of ICU admission: the SAPS II, logistic organ dysfunction system (LODS), systemic inflammatory response syndrome (SIRS) criteria, SOFA score, Charlson Comorbidity Index (CCI), and GCS.

### 2.4. Statistical Analysis

Continuous variables were expressed as mean ± standard deviation or median (interquartile range) and categorical variables as frequencies (percentages). Multivariable logistic regression analysis was employed to evaluate independent predictors of 28‐day mortality in SAE patients, adjusting for potential confounding variables. A generalized additive model (GAM) was applied to explore linear associations and identify optimal thresholds. Kaplan–Meier (KM) survival curves were generated to compare 28‐day survival probabilities between PNI‐stratified groups in both the MIMIC and eICU cohorts. Receiver operating characteristic (ROC) curves were constructed to assess the predictive performance of PNI for mortality. Boxplot analysis was conducted to examine the relationship between PNI values and GCS scores. All statistical analyses were performed using R software, with *p* < 0.05 considered statistically significant.

## 3. Results

### 3.1. Baseline Characteristics

**Figure 1:**
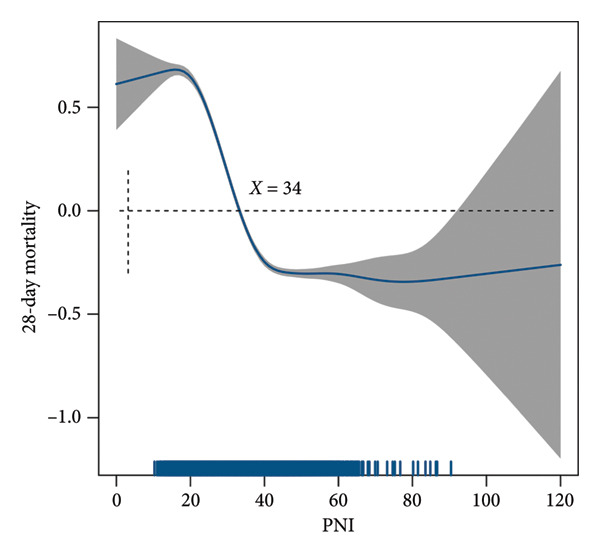
Linear association between PNI and 28‐day mortality in SAE. SAE: sepsis‐associated encephalopathy.

A total of 3202 patients with SAE were included based on predefined criteria (e Figure 1). Participants were stratified into nonsurvivors (*n* = 1062) and survivors (*n* = 2140) according to 28‐day mortality. Nonsurvivors were significantly older and demonstrated profoundly impaired nutritional–immunological status, characterized by substantially lower serum albumin, reduced lymphocyte counts, and critically depressed PNI levels. Furthermore, nonsurvivors exhibited exacerbated organ dysfunction and higher disease severity, evidenced by markedly elevated SOFA, SAPS II, and LODS scores compared to survivors. All baseline variables are presented in Table 1 and eTable 1.

**Table 1:** Baseline characteristics and outcomes of sepsis‐associated encephalopathy patients.

	Original cohort			Match cohort		
	Survival group (*n* = 2140)	Nonsurvival group (*n* = 1062)	*p*	Survival group (*n* = 965)	Nonsurvival group (*n* = 965)	*p*
*Baseline variables*						
Age (years) (median [IQR])	71.00 [59.00,81.00]	68.00 [59.00, 77.00]	< 0.001	69.00 [58.00, 78.00]	68.00 [60.00, 78.00]	0.885
Gender, M (%)	1205 (56.3)	681 (64.1)	< 0.001	594 (61.6)	611 (63.3)	0.452

*Laboratory parameters (median [IQR])*						
Albumin (g/dL)	3.50 [2.90,3.90]	1.60 [1.10,2.10]	< 0.001	3.50 [2.90,4.00]	1.60 [1.10, 2.10]	< 0.001
Lymphocyte (× 10^9^/L)	1.85 [1.42, 2.42]	1.13 [0.73, 1.66]	< 0.001	1.79 [1.38, 2.42]	1.13 [0.73, 1.64]	< 0.001
PNI	44.15 [38.60,49.56]	22.43 [17.85, 28.89]	< 0.001	44.75 [38.70, 50.42]	22.40 [17.85,29.40]	< 0.001

*Critical illness score (median [IQR])*						
CCI	5.00 [3.00, 7.00]	5.00 [3.00, 6.00]	0.055	5.00 [3.00,7.00]	5.00 [3.00, 6.00]	0.055
GCS	8.00 [6.00, 9.00]	3.00 [3.00,3.00]	< 0.001	8.00 [6.00, 9.00]	3.00 [3.00, 3.00]	< 0.001
SOFA	6.00 [4.00, 8.00]	7.00 [6.00, 9.00]	< 0.001	6.00 [4.00, 8.00]	7.00 [6.00, 9.00]	< 0.001
SAPS II	41.00 [32.75, 51.00]	54.00 [40.00, 63.00]	< 0.001	42.00 [33.00, 51.00]	54.00 [40.00, 63.00]	< 0.001
LODS	5.00 [3.00, 8.00]	8.00 [6.00, 9.00]	< 0.001	6.00 [4.00, 8.00]	7.00 [6.00, 9.00]	< 0.001
SIRS	3.00 [2.00, 3.00]	3.00 [2.00, 3.00]	0.005	3.00 [2.00, 3.00]	3.00 [2.00, 3.00]	0.121

*Clinical outcome*						
Vent durations (*n* (%))	1379 (64.4)	924 (87.0)	< 0.001	839 (86.9)	829 (85.9)	0.550
Vasopressin (*n* (%))	214 (10.0)	172 (16.2)	< 0.001	153 (15.9)	151 (15.6)	0.950
Length of stay (median [IQR])	3.90 [1.70, 9.20]	5.30 [2.30, 10.10]	< 0.001	3.80 [1.50, 10.30]	5.20 [2.30, 10.20]	< × 0.001

*Note:* PNI = 10 × albumin (g/dL) + 5 × lymphocyte (× 10^9^/L).

Abbreviations: CCI: Charlson Comorbidity Index; GCS: Glasgow Coma Scale; LODS: Logistic Organ Dysfunction System; SAPS II: Simplified Acute Physiology Score II; SIRS: Systemic Inflammatory Response Syndrome; SOFA: Sequential Organ Failure Assessment.

### 3.2. Association Between PNI and 28‐Day Mortality in SAE

The GAM demonstrated a significant linear relationship between PNI and 28‐day mortality in SAE (Figure 1). A knot value of about 34 was identified, with mortality risk decreasing significantly when PNI exceeded this threshold (Figure 1). Multivariate logistic regression analysis demonstrated that higher PNI was significantly associated with reduced 28‐day mortality in SAE patients (adjusted OR: 0.85, 95% CI: 0.77–0.93, *p* < 0.001) (eTable 2). Consistent findings were observed across alternative analytical approaches: propensity score–matched analysis (OR: 0.87, 95% CI: 0.81–0.93, *p* < 0.001), IPW (OR: 0.82, 95% CI: 0.78–0.86, *p* < 0.001), and doubly robust estimation (OR: 0.81, 95% CI: 0.76–0.86, *p* < 0.001) (Table 2, eFigure 2 and eFigure 3).

**Table 2:** Association between PNI and 28‐day mortality in SAE.

Models	OR	CI		*p*
		2.5%	97.5%	
Multivariate logistic analysis^∗^	0.85	0.77	0.80	< 0.001
Propensity score matching^∗^	0.77	0.81	0.88	< 0.001
Inverse probability weighting^∗^	0.80	0.78	0.81	< 0.001
Doubly robust with all covariates^#^	0.10 exp(coef) [confint] p	0.09 exp(coef) [confint] p	0.12 exp(coef) [confint] p	< 0.001 exp(coef) [confint] p

Abbreviations: PNI, Prognostic Nutritional Index; SAE, sepsis‐associated encephalopathy.

^∗^Analysis was conducted using the continuous variable of PNI.

^#^The doubly robust method requires converting the continuous PNI into a binary categorical variable using a predefined cutoff value for analysis; OR: odds ratio; CI: confidence interval; *p* < 0.05, statistically significant.

### 3.3. Predictive Performance of Prognostic Models for 28‐Day Mortality in SAE

KM survival analysis demonstrated significantly prolonged 28‐day survival in SAE patients with PNI > 34 (Group 2) compared to those with PNI ≤ 34 (Group 1) in both the MIMIC‐IV (*p* < 0.0001; Figure 2(a)) and eICU‐CRD (*p* < 0.0001; Figure 2(b)) cohorts. The survival curves showed clear separation between groups, with Group 2 maintaining higher survival probabilities throughout the 28‐day observation period.

Figure 2KM survival curves of 28‐day mortality in SAE patients and PNI levels. Patients were stratified into two groups based on the PNI, Group 1: PN ≤ 34; Group 2: PNI > 34. (a) Analysis of the MIMIC‐IV cohort; (b) analysis of the eICU‐CRD cohort. KM: Kaplan–Meier; SAE: sepsis‐associated encephalopathy; PNI: Prognostic Nutritional Index; MIMIC‐IV: Medical Information Mart for Intensive Care IV; eICU‐CRD: Electronic Intensive Care Unit Collaborative Research Database.

**Figure:**
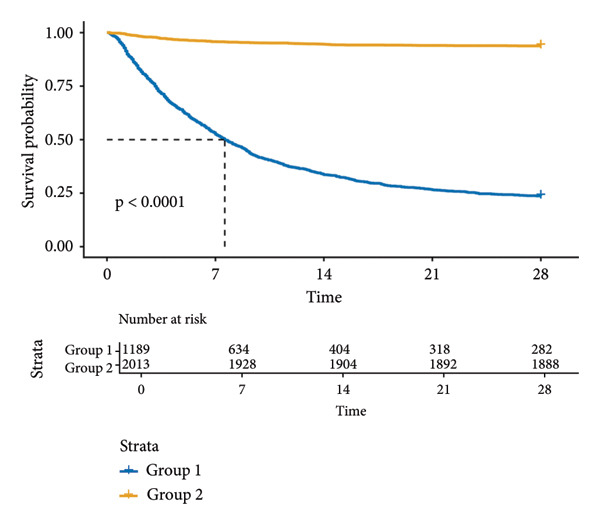
(a)

**Figure:**
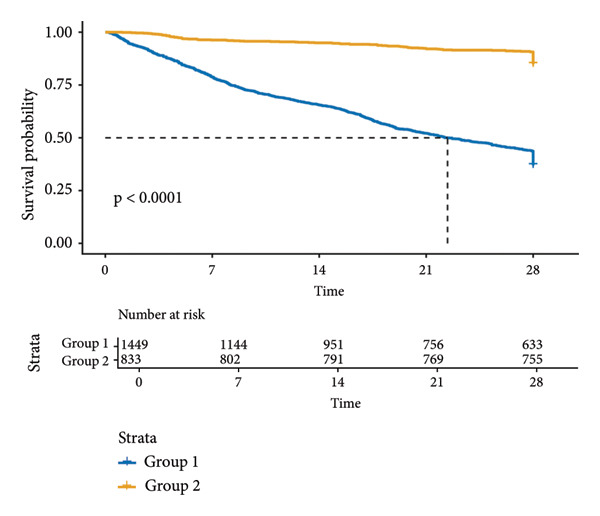
(b)

ROC curve analysis (Figure 3) evaluated four prognostic models for 28‐day mortality prediction in SAE patients: Model 1 (PNI alone) exhibited strong discriminative ability, with AUCs of 0.879 (95% CI: 0.867–0.891) in the MIMIC‐IV cohort (Figures 3(a), 3(c)) and 0.724 (95% CI: 0.707–0.741) in the eICU‐CRD cohort (Figures 3(b), 3(d)). Model 4 (PNI + SOFA + SAPS II) demonstrated superior performance, achieving AUCs of 0.907 (MIMIC‐IV; Figures 3(a), 3(c)) and 0.766 (eICU‐CRD; Figures 3(b), 3(d)), highlighting the added value of combining nutritional status (PNI) with organ dysfunction (SOFA) and physiological severity scores (SAPS II).

Figure 3Predictive performance of prognostic models for 28‐day mortality in SAE. Model 1 = PNI; Model 2 = SOFA; Model 3 = SAPS II; Model 4 = PNI + SOFA + SAPS II. (a) ROC curves for predicting 28‐day mortality in SAE of the MIMIC‐IV cohort; (b) ROC curves for predicting 28‐day mortality in SAE of the eICU‐CRD cohort; (c) comparison of predictive performance for 28‐day mortality in SAE of the MIMIC‐IV cohort; (d) comparison of predictive performance for 28‐day mortality in SAE of the eICU‐CRD cohort. SAE: sepsis‐associated encephalopathy; PNI: Prognostic Nutritional Index; SOFA: Sequential Organ Failure Assessment; SAPS II: Simplified Acute Physiology Score II; ROC: receiver operating characteristic; MIMIC‐IV: Medical Information Mart for Intensive Care IV; eICU‐CRD: Electronic Intensive Care Unit Collaborative Research Database.

**Figure:**
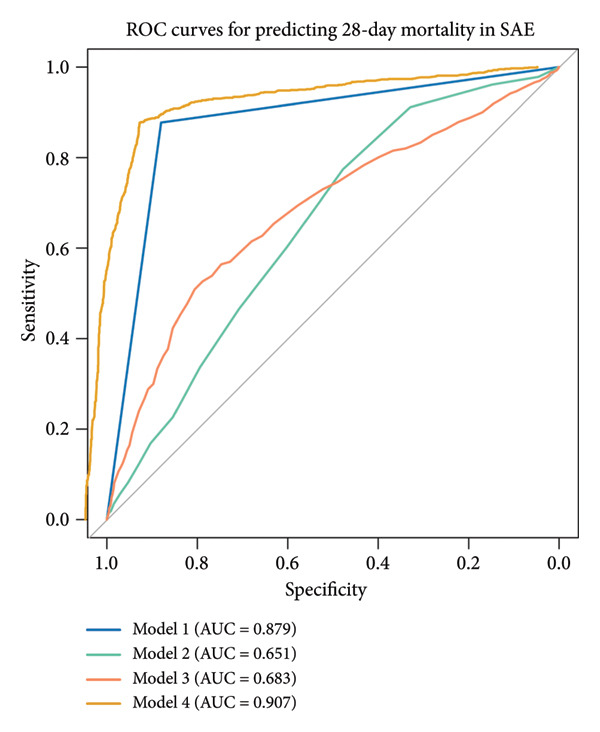
(a)

**Figure:**
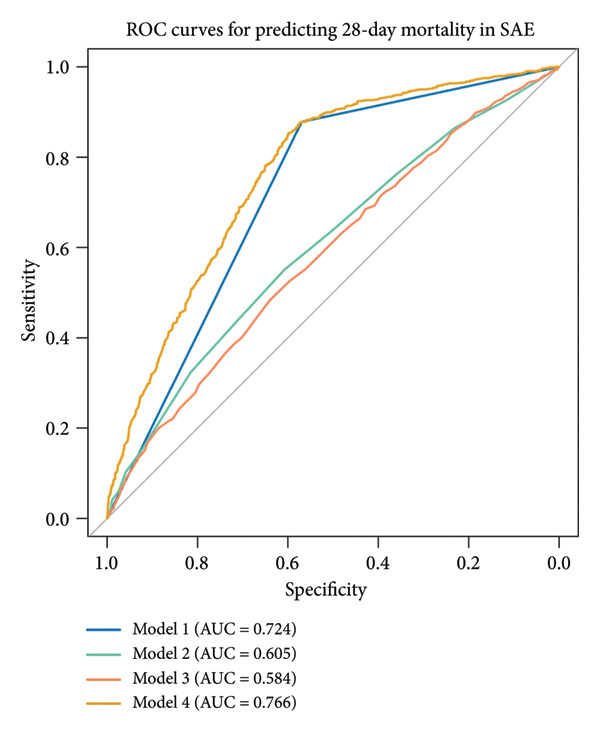
(b)

**Figure:**
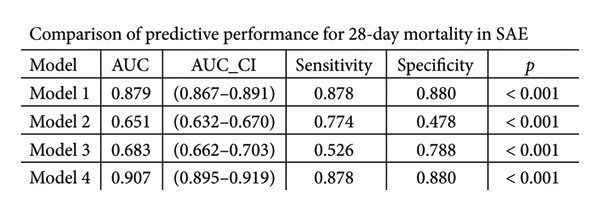
(c)

**Figure:**
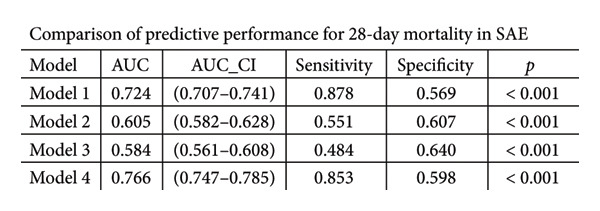
(d)

### 3.4. The Relationship Between PNI and GCS Score

Boxplot analysis revealed a significant inverse correlation between PNI levels and GCS severity categories in both the MIMIC‐IV (*p* < 0.001) and eICU‐CRD (*p* < 0.001) cohorts (Figure 4). As shown in Figure 4(a) (MIMIC‐IV cohort) and Figure 4(b) (eICU‐CRD cohort), PNI values progressively decreased with worsening GCS grade: from 45.6 (41.2–49.8) in Grade 3 (GCS score 13–14) to 38.2 (34.5–42.1) in Grade 1 (GCS score 3–8) (*p* < 0.001). This consistent pattern across both databases demonstrates that better nutritional status (higher PNI) is associated with higher levels of consciousness (better GCS scores).

Figure 4The relationship between PNI and GCS grade. GCS Grade 1: GCS score 3–8; GCS Grade 2: GCS score 9–12; GCS Grade 3: GCS score 13–14. (a) Analysis of the MIMIC‐IV cohort; (b) analysis of the eICU‐CRD cohort. PNI: Prognostic Nutritional Index; GCS: Glasgow Coma Scale; MIMIC‐IV: Medical Information Mart for Intensive Care IV; eICU‐CRD: Electronic Intensive Care Unit Collaborative Research Database.

**Figure:**
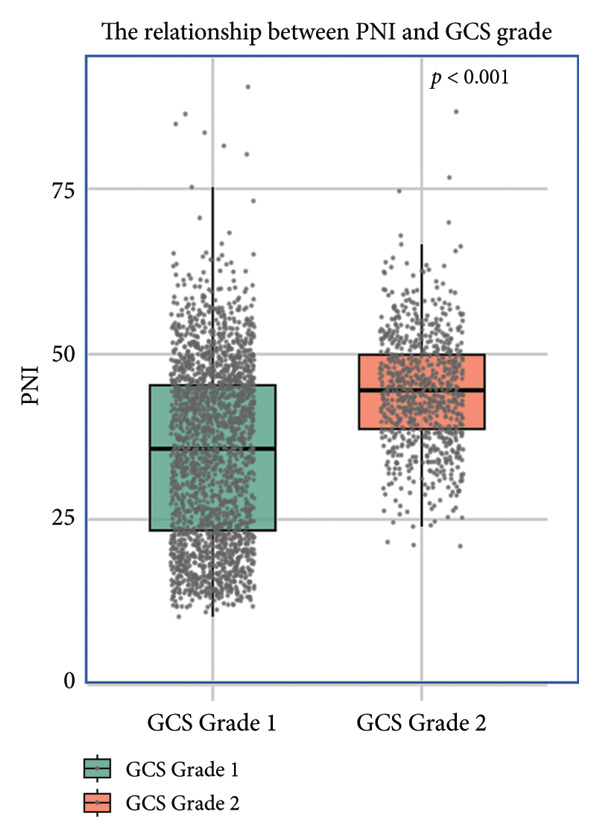
(a)

**Figure:**
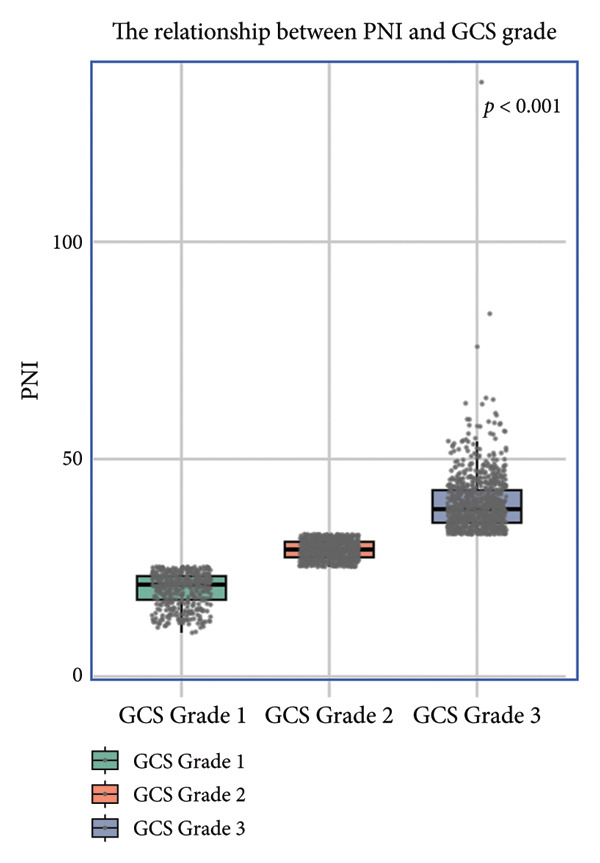
(b)

## 4. Discussion

Our findings demonstrate that SAE patients had a 28‐day mortality rate of 33.2%. The PNI was a significant predictor of 28‐day mortality in patients with SAE, and its predictive performance was superior to that of the SOFA and SAPS II scores, with PNI levels showing a linear correlation with increased mortality risk. Stratified analysis shows that progressively lower PNI levels were associated with worsening degrees of consciousness impairment.

The multicenter analysis demonstrates unabated high 28‐day mortality rates among SAE patients, consistent with established clinical evidence [2, 3]. Despite substantial research efforts, the current lack of sensitive prognostic biomarkers impedes early risk stratification and targeted interventions for SAE. Our findings reinforce the critical need to identify reliable indicators that can dynamically predict outcomes and guide therapeutic escalation in this vulnerable population.

We established the PNI as a novel independent predictor of SAE mortality, outperforming conventional severity scores (SOFA/SAPS II) in both discrimination and clinical utility. While the optimal threshold (PNI = 34) robustly stratified mortality risk across cohorts for clinical decision‐making, the GAM revealed a more nuanced continuous dose–response relationship—each 1‐point decrease in PNI below 34 was associated with a 3.7% incremental mortality risk increase, demonstrating that the binary categorization, though operationally useful, underrepresents the true gradient of risk. This linear association persisted even above the threshold, suggesting PNI’s predictive value extends across its entire physiological range as a dynamic continuum reflecting cumulative nutritional–inflammatory burden. The mechanisms underlying PNI’s predictive value in SAE likely involve dual pathways: (1) Albumin was associated with SAE and was supported by medium‐ to high‐quality evidence [6]. Use of albumin decreased the risk of sepsis‐associated delirium [5]. Hypoalbuminemia exacerbates blood–brain barrier disruption via oxidative stress and endothelial dysfunction. (2) Lymphocytopenia reflects impaired immunomodulation, worsening neuroinflammation. The lymphocyte population comprises three principal immunophenotypically distinct subsets: T lymphocytes (T cells), B lymphocytes (B cells), and natural killer cells (NK cells). T cells constitute a major subset of lymphocytes, representing one of the principal cellular components within the lymphoid lineage. V‐domain immunoglobulin suppressor of T‐cell activation (VISTA) has emerged as a crucial player in the pathogenesis of neurological disorders [17]. CD86 in CD3 + CD56 + natural killer T (NKT) cells is an independent risk factor of SAE [18]. Mechanistically, PNI integrates hypoalbuminemia‐driven blood–brain barrier disruption and lymphopenia‐mediated neuroinflammation—core pathways in SAE pathogenesis [19–23]. We therefore recommend serial PNI monitoring in SAE management, with prompt nutritional–immunomodulatory therapy when PNI falls below 34.

Stratified analysis demonstrated a significant correlation between higher PNI and improved GCS scores, indicating PNI’s capacity to reflect SAE‐induced consciousness impairment severity. While our clinical findings robustly establish this association, we acknowledge that the underlying pathophysiological mechanisms require further molecular‐level validation through targeted experimental studies. The observed relationship may be mediated through both albumin‐dependent and lymphocyte‐mediated pathways [21]: Albumin’s neuroprotective effects may attenuate oxidative neuronal injury by scavenging free radicals and stabilizing blood–brain barrier integrity, while lymphocyte subsets (particularly T cells and NKT cells expressing immunoregulatory markers like CD86) may modulate neuroinflammation through cytokine regulation and microbial clearance [3, 24]. Emerging evidence suggests that VISTA‐mediated immunomodulation in microglia and lymphocyte trafficking across the blood–brain barrier could represent plausible mechanistic links between PNI components and neurological outcomes [25, 26]. However, definitive confirmation of these pathways awaits future investigations combining cellular models with clinical biomarker studies. Clinicians should, nevertheless, utilize PNI trends to anticipate neurological trajectory and personalize neuroprotective strategies (e.g., antioxidant supplementation and infection source control) in comatose SAE patients, while remaining cognizant of the need for further mechanistic clarification.

The PNI alone (Model 1) exhibited strong discriminative ability in the original MIMIC‐IV cohort (AUC = 0.879). However, the predictive performance was notably lower in the independent validation eICU‐CRD cohort (AUC = 0.724). The superior predictive model (PNI + SOFA + SAPS II) also showed a significant drop in performance, from an AUC of 0.907 in MIMIC‐IV to 0.766 in eICU‐CRD3. The observed performance variation between cohorts primarily reflects fundamental differences in database architectures. As a single‐center registry, MIMIC‐IV provides inherently more standardized patient data with uniform diagnostic criteria and measurement protocols. In contrast, the eICU‐CRD’s multicenter design introduces unavoidable heterogeneity in case definitions, laboratory methodologies, and clinical documentation practices—factors known to attenuate biomarker performance in validation studies. Notably, despite these inherent challenges of multicenter validation, the PNI maintained clinically meaningful discrimination (AUC > 0.70) in the eICU‐CRD cohort, outperforming conventional severity scores (SOFA AUC = 0.682, SAPS II AUC = 0.704). This robustness across distinct database paradigms substantiates PNI’s reliability as a generalizable prognostic indicator for SAE.

The observed differential mortality associations with *Klebsiella pneumoniae* (OR 0.27) and *Escherichia coli* (OR 4.99) in multivariate analysis—despite comparable baseline prevalence—suggest potential pathogen‐specific interactions with PNI’s components. Gram‐negative pathogens like *E. coli* may amplify PNI’s prognostic value through synergistic albumin–endotoxin binding and lymphocyte‐mediated clearance mechanisms, whereas the neuroprotective effects of albumin may be particularly critical in *K. pneumoniae* infections given its propensity for hyperinflammatory responses. This aligns with our finding that PNI integrates both inflammatory (albumin) and immunological (lymphocyte) pathways, which are differentially engaged depending on microbial virulence factors. While our current analysis did not stratify by infection site due to sample size constraints, the pathogen‐specific mortality patterns imply that PNI’s predictive performance may be modulated by the underlying microbiology—a hypothesis requiring future investigation with targeted microbial subtyping. Importantly, PNI remained an independent predictor after adjusting for pathogen type, confirming its broad applicability while highlighting opportunities to refine its interpretation based on causative organisms in clinical practice.

## 5. Limitations

Several study limitations warrant acknowledgment. First, the retrospective design inherently carries potential selection bias, notwithstanding comprehensive statistical adjustment. Second, while validated across two independent databases, the generalizability of the established PNI threshold (34) requires prospective verification. Third, residual confounding from unmeasured variables (e.g., nutritional supplementation regimens and underlying comorbidities) may potentially influence clinical outcomes. Fourth, despite robust adjustments for measurable confounders, the retrospective design precludes causal inferences, and unmeasured variables—particularly nutritional supplementation regimens (e.g., timing, route, and dose of albumin or immunonutrition) and granular comorbidity data (e.g., severity of underlying conditions)—may residually confound the observed PNI–mortality association. While PNI reflects baseline status, its predictive accuracy could be influenced by undocumented therapeutic interventions that alter albumin or lymphocyte trajectories. Future prospective studies should systematically track nutritional interventions (timing, type, and duration) and comorbidities to disentangle their effects from baseline PNI. Finally, the pathophysiological mechanisms mediating the observed PNI–GCS association remain hypothetical and necessitate further mechanistic investigation.

## 6. Conclusions

This multicenter study identified the PNI as a robust and independent predictor of 28‐day mortality in patients with SAE, demonstrating an optimal prognostic threshold of 34. The consistent predictive performance across diverse analytical methodologies and independent validation cohorts substantiates its clinical utility for risk stratification in SAE management.

**Nomenclature**
CCI: Charlson Comorbidity Index
CI: Confidence interval
eICU‐CRD: Electronic Intensive Care Unit Collaborative Research Database
GAM: Generalized additive model
GCS: Glasgow Coma Scale
INR: International Normalized Ratio
IPW: Inverse probability weighting
KM: Kaplan–Meier
LODS: Logistic organ dysfunction system
MIMIC‐IV: Medical Information Mart for Intensive Care IV
OR: Odds ratio
PNI: Prognostic Nutritional Index
PSM: Propensity score matching
PT: Prothrombin time
PTT: Partial thromboplastin time
ROC: Receiver operating characteristic
SAE: Sepsis‐associated encephalopathy
SAPS II: Simplified Acute Physiology Score II
SIRS: Systemic inflammatory response syndrome
SMD: Standardized mean difference
SOFA: Sequential Organ Failure Assessment
SQL: Structured Query Language

## Ethics Statement

The MIMIC‐IV and eICU‐CRD databases were approved by the institutional review boards of the Massachusetts Institute of Technology and Beth Israel Deaconess Medical Center. All methods were carried out in accordance with relevant guidelines and regulations. The informed consent requirement was waived by these institutional review boards because the project did not impact clinical care, and all patient confidential information was anonymized.

## Consent

Please see the Ethics Statement.

## Disclosure

A previous version of this manuscript was published as a preprint on Research Square, available at: https://www.researchsquare.com/article/rs-8037637/v1 [27].

## Conflicts of Interest

The authors declare no conflicts of interest.

## Author Contributions

Dr Lina Zhao had full access to all of the data in the study and takes responsibility for the integrity of the data and the accuracy of the data analysis. Concept and design: Lina Zhao, Chao Qi, and Keliang Xie. Acquisition, analysis, or interpretation of data: all authors. Drafting of the manuscript: Lina Zhao, Chao Qi, and Keliang Xie. Critical review of the manuscript for important intellectual content: all authors. Data collection and statistical analysis: Chao Qi, Yuehao Shen, Haiying Liu, Xuguang Li, Dongxue Huang, and Qinghe Yan. Obtained funding: Lina Zhao. Administrative, technical, or material support: Yun Li, Yuehao Shen, Haiying Liu, Xuguang Li, Dongxue Huang, and Qinghe Yan. Supervision: Yun Li and Keliang Xie. Lina Zhao, Chao Qi, and Yun Li contributed equally to this study.

## Funding

This work was supported by joint funds of the Natural Science Foundation of Tianjin (No. 25JCLMJC00350).

## Supporting Information

eTable 1: Baseline characteristics of sepsis‐associated encephalopathy patients. eTable 2: Multivariable logistic analysis of factors associated with 28‐day mortality in patients with sepsis‐associated encephalopathy. eFigure 1: Flowchart for patient selection.

## Supporting information


**Supporting Information** Additional supporting information can be found online in the Supporting Information section.

## Data Availability

The original data can be made available upon reasonable request from the corresponding author. The study utilized data from MIMIC‐IV and eICU‐CRD databases.
