# Supplementary material for: Prognostic Nutritional Index as a Novel Biomarker for Predicting Prognosis in Sepsis‐Associated Encephalopathy: A Multicenter Retrospective Cohort Study
Source: Emerg Med Int. 2026 Jan 7;2026:4486190. doi: 10.1155/emmi/4486190 (PMC12779611; doi:10.1155/emmi/4486190)
Supplement: Supplementary file 1 — Supporting Information Additional supporting information can be found online in the Supporting Information section. [file EMMI-2026-4486190-s001.doc]

| **eTable 1 Baseline characteristics of sepsis-associated encephalopathy patients** | | | | | | |
| --- | --- | --- | --- | --- | --- | --- |
|  | **Original cohort** | | | **Match cohort** | | |
|  | Survival group (*n* = 2140) | Non-survival group(*n* = 1062) | *P* | Survival group (*n* = 965) | Non-survival group(*n* = 965) | *P* |
| **Co-existing illness (n(%))** | | | | | | |
| Hypertension | 276(12.9) | 168 ( 15.8) | 0.028 | 140(14.5) | 144 ( 14.9) | 0.847 |
| Diabetes | 543(25.4) | 221 ( 20.8) | 0.005 | 252(26.1) | 200 ( 20.7) | 0.006 |
| **Site of infection (n(%))** | | | | | | |
| Urinary | 152 ( 7.1) | 42 ( 4.0) | < 0.001 | 53 ( 5.5) | 37 ( 3.8) | 0.105 |
| Lung | 146 ( 6.8) | 61 ( 5.7) | 0.275 | 58 ( 6.0) | 54 ( 5.6) | 0.77 |
| Catheter-based | 55 ( 2.6) | 19 ( 1.8) | 0.208 | 26 ( 2.7) | 18 ( 1.9) | 0.286 |
| Soft tissue and skin | 76 ( 3.6) | 26 ( 2.4) | 0.117 | 37 ( 3.8) | 21 ( 2.2) | 0.046 |
| Abdominal cavity | 62 ( 2.9) | 22 ( 2.1) | 0.208 | 31 ( 3.2) | 21 ( 2.2) | 0.206 |
| **Microbiology type (n (%))** | | | | | | |
| Acinetobacter baumannii | 13 ( 0.6) | 4 ( 0.4) | 0.557 | 1 ( 0.1) | 3 ( 0.3) | 0.617 |
| Klebsiella pneumoniae | 188 ( 8.8) | 60 ( 5.6) | 0.002 | 78 ( 8.1) | 58 ( 6.0) | 0.091 |
| Escherichia Coli | 332 (15.5) | 114 ( 10.7) | < 0.001 | 122(12.6) | 111 ( 11.5) | 0.485 |
| Pseudomonas aeruginosa | 110 ( 5.1) | 40 ( 3.8) | 0.100 | 48( 5.0) | 35 ( 3.6) | 0.178 |
| Staphylococcus aureus | 612(28.6) | 215 ( 20.2) | < 0.001 | 267(27.7) | 191 ( 19.8) | < 0.001 |
| **Laboratory parameters (median [IQR])** | | | | | | |
| Hemoglobin (g/dL) | 9.70[8.20, 11.50] | 9.30[8.03,10.80] | < 0.001 | 9.30[8.00, 10.90] | 9.30 [8.10, 10.80] | 0.918 |
| Platelet ( × 10^9^ /L) | 177.00[120.00,242.00] | 139.00[103.00, 194.00] | < 0.001 | 148.00[106.00,203.00] | 142.00[104.00, 200.00] | 0.39 |
| White blood cell (× 10^9^ /L) | 13.00 [9.40, 17.40] | 14.45 [10.70, 18.90] | < 0.001 | 13.90 [10.20, 18.30] | 14.50 [10.70, 18.80] | 0.05 |
| Blood urea nitrogen (mmol/L) | 21.00[15.00, 29.00] | 20.00 [15.00, 27.00] | 0.01 | 20.00[14.00, 28.00] | 20.00 [15.00, 27.00] | 0.667 |
| Creatinine (mg/dL) | 1.10[0.80, 1.60] | 1.00 [0.80, 1.50] | 0.271 | 1.10[0.80, 1.60] | 1.00[0.80,1.50] | 0.133 |
| Glucose (mg/dL) | 143.00[117.00,180.00] | 134.00[112.00, 169.00] | < 0.001 | 141.00[116.00,177.00] | 134.00[112.00, 170.00] | 0.019 |
| Sodium (mmol/L) | 140.00[138.00,143.00] | 140.00[138.00, 142.00] | 0.061 | 140.00[138.00,143.00] | 140.00[138.00, 142.00] | 0.263 |
| INR | 1.40 [1.20, 1.60] | 1.40 [1.20, 1.60] | < 0.001 | 1.40 [1.20, 1.70] | 1.40 [1.20, 1.60] | 0.816 |
| PT (s) | 14.90 [12.80, 18.00] | 15.50 [13.70, 18.00] | < 0.001 | 15.60 [13.30, 18.10] | 15.40 [13.50, 18.00] | 0.964 |
| PTT (s) | 33.90 [28.90, 45.00] | 35.10 [29.92, 47.18] | < 0.001 | 35.80 [29.90, 47.20] | 34.60 [29.70, 46.00] | 0.406 |
| Lactates (mmol/L) | 1.70 [1.20, 2.20] | 1.80 [1.30, 2.60] | < 0.001 | 1.80 [1.30, 2.60] | 1.80 [1.30, 2.50] | 0.184 |
| pH | 7.43 [7.40, 7.46] | 7.44 [7.41, 7.48] | < 0.001 | 7.43 [7.41, 7.47] | 7.44 [7.41, 7.48] | 0.171 |
| PaO_2_ (mmHg) | 258.00 [123.75, 288.00] | 329.50 [212.50, 425.00] | < 0.001 | 269.00 [213.00, 409.00] | 306.00 [196.00, 411.00] | 0.723 |
| PaCO_2_ (mmHg) | 35.00 [33.00, 39.00] | 34.00 [30.00, 36.00] | < 0.001 | 35.00 [31.00, 37.00] | 34.00 [31.00, 37.00] | 0.382 |
| Vital signs, (median [IQR]) | | | | | | |
| Heart rate(bpm) | 105.57 (20.63) | 102.03 (19.34) | < 0.001 | 103.19 (19.72) | 102.36 (19.63) | 0.355 |
| Systolic blood pressure (mmHg) | 90.00 [80.00, 101.00] | 88.00 [79.00, 96.00] | < 0.001 | 87.00 [79.00, 98.00] | 88.00 [79.00, 96.00] | 0.714 |
| Diastolic blood pressure (mmHg) | 45.00[39.00, 52.00] | 45.00 [39.00, 51.00] | 0.145 | 45.00 [38.00, 51.00] | 45.00 [39.00, 51.00] | 0.807 |
| Mean arterial pressure (mmHg) | 58.00 [51.00, 66.00] | 58.00 [52.00, 64.00] | 0.068 | 58.00 [51.00, 64.00] | 58.00[52.00,64.00] | 0.848 |
| Respiratory rate (bpm) | 28.00 [24.00, 32.00] | 27.00 [23.00, 31.00] | < 0.001 | 27.00 [23.50, 31.00] | 27.00 [23.00, 31.00] | 0.985 |
| Temperature （℃） | 36.70 [36.60, 37.10] | 36.70 [36.60, 37.10] | 0.06 | 36.70[36.60, 37.10] | 36.70 [36.60, 37.10] | 0.109 |
| SpO_2_ | 98.00[97.00, 98.00] | 98.00[97.00,98.00] | 0.005 | 98.00[97.00, 98.00] | 98.00 [97.00, 98.00] | 0.529 |

INR: international normalized ratio; PT: prothrombin time; PTT: partial thromboplastin time; pH: potential of hydrogen; PaO_2_: partial pressure of oxygen in arterial blood; PaCO₂: arterial partial pressure of carbon dioxide; SpO₂: peripheral oxygen saturation

| **eTable 2** Multivariable Logistic analysis of factors associated with 28-day mortality in patients with sepsis-associated encephalopathy | | | |
| --- | --- | --- | --- |
|  | OR | 95%CI | *P* |
| Age | 1.06 | 1.03-1.10 | < 0.001 |
| Gender | 1.31 | 0.59-2.85 | 0.5 |
| PNI | 0.85 | 0.81-0.88 | < 0.001 |
| **Critical illness score** | | | |
| GCS | 0 | 0.00-0.00 | 0.979 |
| SOFA | 0.93 | 0.79-1.09 | 0.363 |
| SAPS II | 0.98 | 0.94-1.03 | 0.401 |
| LODS | 1.13 | 0.90-1.41 | 0.291 |
| SIRS | 1.25 | 0.82-1.90 | 0.298 |
| Ventdurations | 2.8 | 0.98-7.70 | 0.049 |
| Length of stay | 1.1 | 1.03-1.20 | 0.008 |
| **Co-existing illness** | | | |
| Hypertension | 1 | 0.36-1.25 | 0.997 |
| Diabetes | 0.62 | 0.50-1.50 | 0.281 |
| Urinary infection | 2 | 0.24-48.89 | 0.586 |
| **Microbiology type** | | | |
| Klebsiella pneumoniae | 0.27 | 0.08-0.99 | 0.039 |
| Escherichia Coli | 4.99 | 1.17-30.39 | 0.049 |
| Staphylococcus aureus | 0.54 | 0.24-1.25 | 0.139 |
| **Laboratory parameters** | | | |
| Hemoglobin | 1.1 | 0.89-1.37 | 0.373 |
| Platelet | 1 | 0.99-1.00 | 0.101 |
| White blood cell | 1.03 | 0.99-1.08 | 0.23 |
| Blood urea nitrogen | 0.98 | 0.93-1.02 | 0.3 |
| Glucose | 1 | 1.00-1.01 | 0.808 |
| INR | 8.1 | 0.00-1.33 | 0.603 |
| PT | 1.04 | 0.97-2.17 | 0.91 |
| PTT | 0.99 | 0.98-1.01 | 0.452 |
| PaO_2_ | 1 | 0.99-1.00 | 0.094 |
| PaCO_2_ | 0.98 | 0.94-1.01 | 0.166 |
| **Vital signs** | | | |
| Heart rate | 1.02 | 0.99-1.04 | 0.22 |
| Systolic blood pressure | 1.02 | 1.00-1.04 | 0.087 |
| Respiratory rate | 1 | 0.95-1.06 | 0.995 |

PNI = 10 × Albumin (g/dL) + 5 × Lymphocyte (× 10⁹/L); GCS: Glasgow Coma Scale;SOFA: Sequential Organ Failure Assessment; SAPS II: Simplified Acute Physiology Score II; LODS: Logistic organ dysfunction system; SIRS: Systemic Inflammatory Response Syndrome; INR: international normalized ratio; PT: prothrombin time; PTT: partial thromboplastin time; pH: potential of hydrogen; PaO_2_: partial pressure of oxygen in arterial blood; PaCO_2_: arterial partial pressure of carbon dioxide; OR: odds ratio; CI: confidence interval

**eFigure 1** Flow chart for patient selection


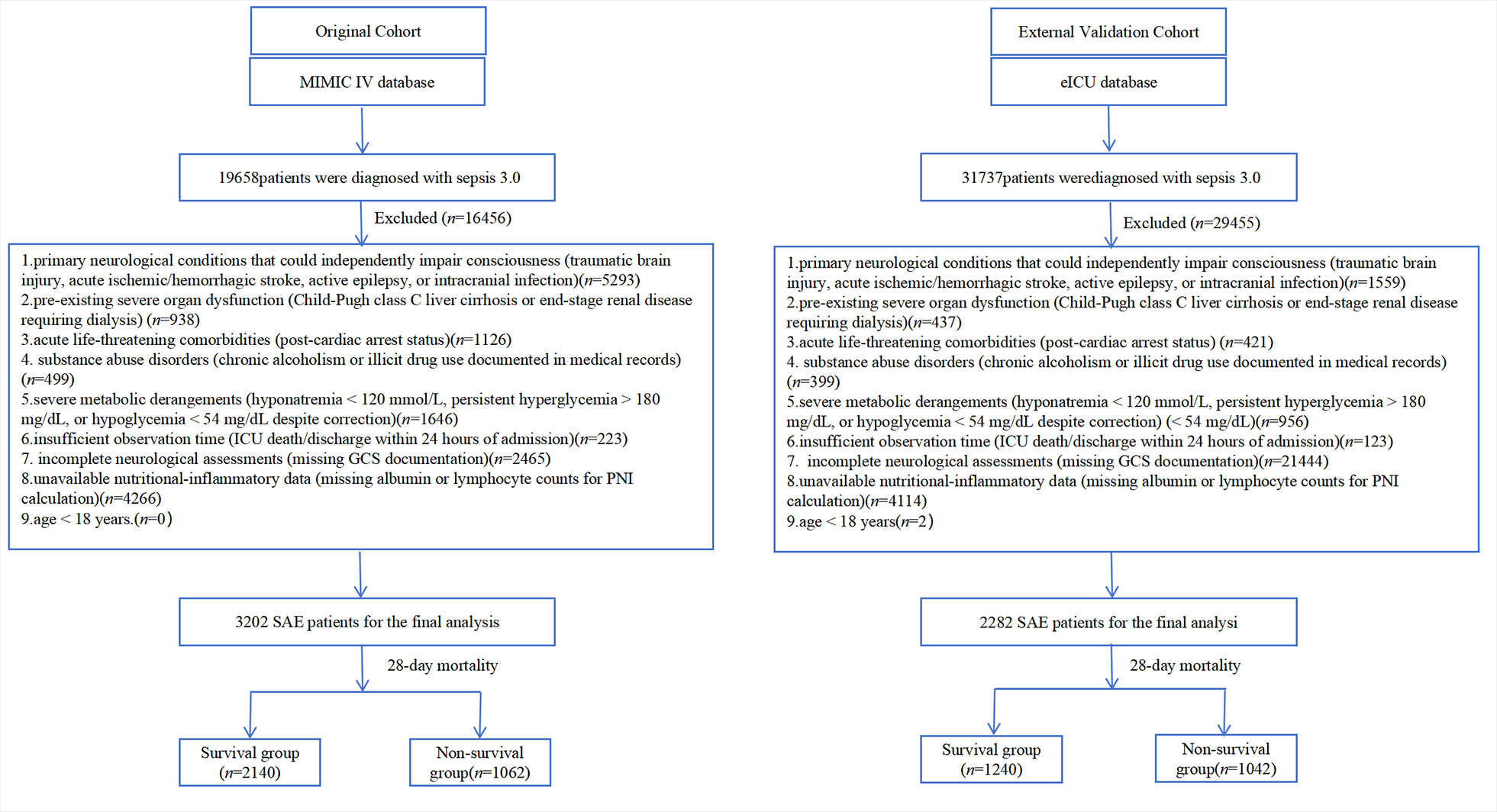


**eFigure 2** The SMD of propensity-matched


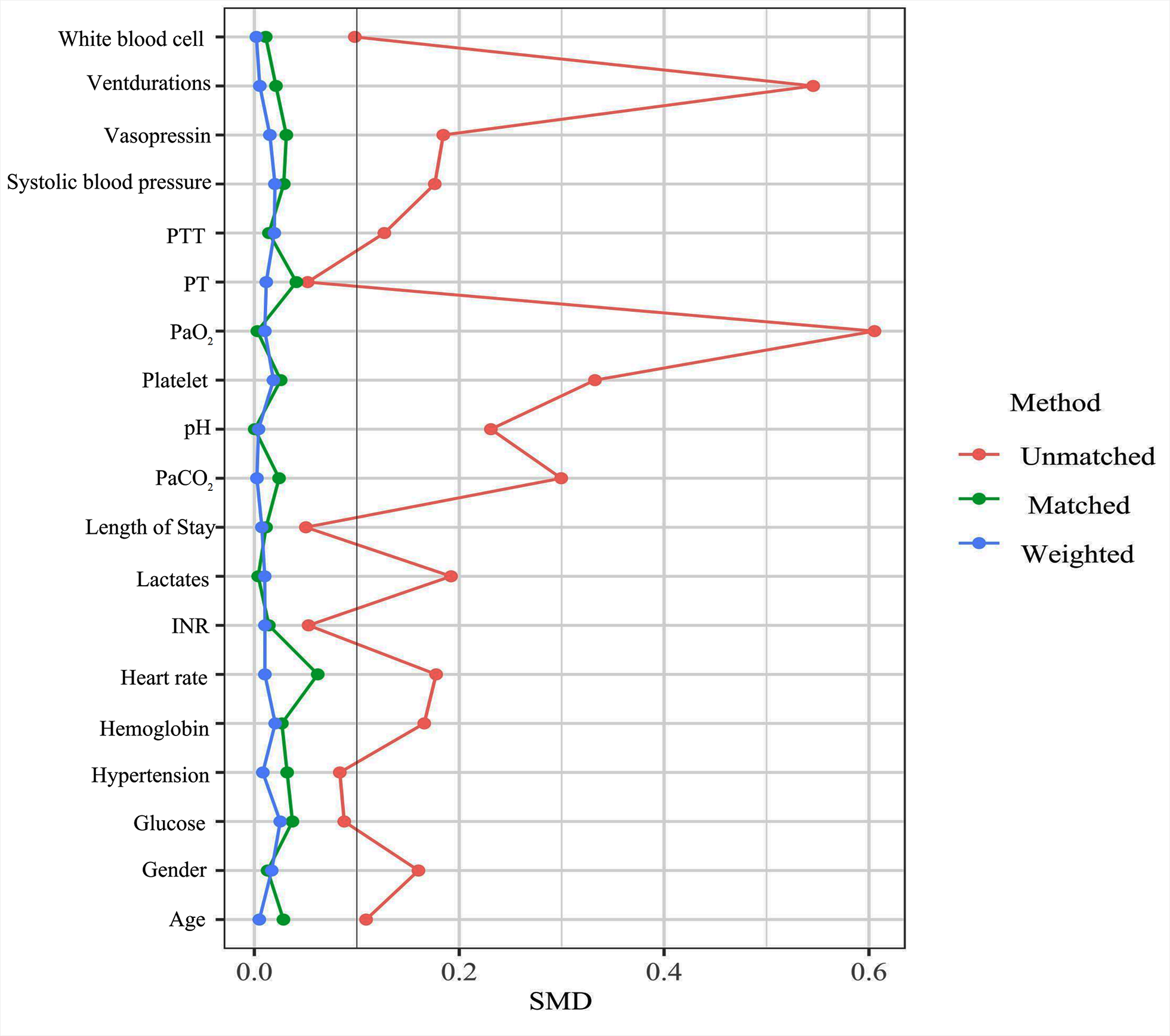


PTT: partial thromboplastin time; PT: prothrombin time; INR: international normalized ratio; PaO₂: partial pressure of oxygen in arterial blood; pH: potential of hydrogen; PaCO₂: arterial partial pressure of carbon dioxide; INR: International normalized ratio.

**eFigure 3** Forest plot of multivariable Logistic regression analysis influencing the primary outcomes of SAE patients.

PNI: Prognostic Nutritional Index; OR: odds ratio; CI: confidence interval
